# Redirecting an Anticancer to an Antibacterial Hit Against Methicillin-Resistant *Staphylococcus aureus*

**DOI:** 10.3389/fmicb.2019.00350

**Published:** 2019-02-25

**Authors:** Hye-Jeong Jang, In-Young Chung, Changjin Lim, Sungkyun Chung, Bi-o Kim, Eun Sook Kim, Seok-Ho Kim, You-Hee Cho

**Affiliations:** Department of Pharmacy, College of Pharmacy and Institute of Pharmaceutical Sciences, CHA University, Seongnam, South Korea

**Keywords:** MRSA, gram-positive, antibacterials, drug repurposing, drug redirecting

## Abstract

YM155 is a clinically evaluated anticancer with a fused naphthoquinone-imidazolium scaffold. In this study, we demonstrated that based on weak or cryptic antibacterial activity of YM155 against methicillin-resistant *Staphylococcus aureus* (MRSA) (MIC of 50 μg/ml), some congeneric compounds with short alkyl chains (e.g., c5 with a hexyl chain) at the N3 position of the scaffold, displayed more potent antibacterial activity against MRSA (MIC of 3.13 μg/ml), which is in a clinically achievable range. Their antibacterial activity was evident against Gram-negative bacteria, only in the presence of the outer membrane-permeabilizing agent, polymyxin B. The antibacterial efficacy of c5 was confirmed using the *Drosophila* systemic infection model. We also characterized five spontaneous c5-resistant MRSA mutants that carry mutations in the *ubiE* gene, for quinone metabolism and respiratory electron transfer, and subsequently exhibited reduced respiration activity. The antibacterial activity of c5 was compromised either by an antioxidant, *N*-acetylcysteine, or in an anaerobic condition. These suggest that the antibacterial mechanism of c5 involves the generation of reactive oxygen species (ROS), presumably during respiratory electron transport. This study provides an insight into “drug redirecting,” through a chemical modification, based on an ROS-generating pharmacophore.

## Introduction

Since antibiotic therapy was introduced into clinical practice, bacterial pathogens have been developing antibiotic resistance more rapidly. This resistance reduces or eliminates the effectiveness of the antibiotic regimen. In addition, opportunistic pathogens with intrinsic resistance to multiple antibiotics have become emerging problems in public health. In developed countries, a group of bacterial species that have acquired multiple drug resistances are often referred to as the ESKAPE pathogens, aptly named for their ability to “escape” the effects of currently available antibacterial drugs (Rice, 2008). *Staphylococcus aureus* is noteworthy among those pathogens as it is a very versatile Gram-positive bacterium, whose antibiotic resistance has been of great concern, particularly in hospital environments where it is often the cause of post-surgical wound infections (Cohen, 1992; Tomasz, 1994; Engemann et al., 2003). Infections with *S. aureus* have become increasingly difficult to treat due to the emergence and rapid spread of methicillin-resistant *S. aureus* (MRSA), which are important nosocomial pathogens associated with increased morbidity and mortality worldwide (Lowy, 1998; Diekema et al., 2001). MRSA has become increasingly resistant to multiple classes of antibiotics including not only β-lactams, but also macrolides, quinolones, and even vancomycin (Leclercq, 2002; Lowy, 2003). Because community-associated MRSA infections are becoming more and more common (Mediavilla et al., 2012), there is an urgent need to develop antibacterial agents with features aimed to effectively target MRSA infections.

Although the search for antibacterials with novel scaffolds is of utmost importance at this juncture, mining and/or repurposing based on chemically modified bioactive compounds is considered an attractive proposition. Fused imidazolium analogs are known as anticancer agents, in large part due to their involvement in the reactive oxygen species (ROS)-mediated enhancement of apoptotic functions, although the additional and detailed mode of action remains elusive (Ho et al., 2015). Among them, YM155 (sepantronium bromide) [1-(2-methoxyethyl)-2-methyl-3-(pyrazin-2-ylmethyl)-4,9-dioxo-4,9-dihydro-1*H*-naphtho(2,3-*d*) imidazolium bromide] was identified by a high-throughput screening of chemical libraries, to screen for inhibitors of the expression of an anti-apoptotic protein, survivin, whose expression is increased in most solid tumors (Nakahara et al., 2007). Preclinical studies using YM155 showed inhibition of survivin at both the mRNA and protein levels and exhibited anticancer activity in mouse models (Nakahara et al., 2007).

In this study, we have identified cryptic or weak antibacterial activity of an anticancer, YM155, against MRSA and enhanced the activity through chemical modification at its N3 position. Its congeners with short alkyl chains, instead of the pyrazin-2-ylmethyl moiety, displayed more potent antibacterial activity. Their inhibitory activity is selective toward Gram-positive bacteria, due to the permeability barrier in Gram-negative bacteria and presumably in mammalian cells. This strategy conceptualizes “drug redirecting,” which may be distinct from “drug repurposing” in that it involves drug modification to change/redirect the cellular targets and the subsequent indications.

## Materials and Methods

### Bacterial Strains and Culture Conditions

The bacterial strains used in this study are listed in (Supplementary Table S1). *Pseudomonas aeruginosa*, *Escherichia coli*, *Klebsiella pneumoniae*, *Bacillus subtilis*, *S. aureus* strains were grown at 37°C using Luria-Bertani (LB) (1% tryptone, 0.5% yeast extract, and 1% NaCl) broth, Mueller-Hinton (MH) broth, and M9-glucose minimal medium (1.2% Na_2_HPO_4_, 0.3% KH_2_PO_4_, 0.05% NaCl, 0.1% NH_4_Cl, 2 mM MgSO_4_, 0.1 mM CaCl_2_, and 0.4% glucose) or on 2% Bacto-agar solidified LB plates. Overnight-grown cultures were used as inoculum (1.6 × 10^7^ CFU/ml) into fresh LB broth and grown at 37°C in a shaking incubator until the logarithmic (OD_600_ = 1.0) phase, and then the cell cultures were used for the experiments described herein. For anaerobic growth, bacteria were grown in an LB medium in an anaerobic jar with AnaeroPack (MGC).

### Synthesis of YM155 and Its Congeners

The fused imidazolium analogs were synthesized and purified as described previously (Kuo et al., 1996; Ho et al., 2015). All reagents and chemicals were obtained from Sigma-Aldrich and Merck, unless otherwise specified. YM155 was purchased from Aladdin Industrial Corporation. UV spectra were recorded on a Jasco spectrophotometer equipped with a Peltier temperature control unit. The synthesized compounds were dissolved in dimethyl sulfoxide (DMSO).

### Determination of Minimal Inhibitory Concentration (MIC)

Minimal inhibitory concentrations for YM155 congeners were determined in MH broth by the broth microdilution method, using standard microbiological procedures according to NCCLS guidelines, as per document no. M07-A8 (2012). The medium, with a 2-fold serial dilution of each compound in the MH broth, was subjected to inoculation with the indicated bacterial strains (5 × 10^5^ CFU/ml) that had been grown at 37°C to the logarithmic growth phase (OD_600_ = 1.0) and then incubated at 37°C on a rotatory shaker. The MIC values were recorded as the lowest concentration of the compound at which no signs of growth were observed, based on the OD_600_ value of less than 0.05 after 18 h of incubation. The MIC values were confirmed by three independent experiments. Methicillin and gentamicin were used as the control antibiotics.

### Measurement of Antibacterial Activity

The sensitivity of bacterial cells was evaluated by a spotting assay. The 10-fold serial dilutions (3 μl) of the cell cultures in the LB broth were spotted onto an LB agar plate containing chemicals such as YM155 and its analogs or antibiotics (methicillin or gentamicin), at the indicated concentrations, to enumerate the survivor bacteria. The plates were incubated overnight at 37°C. For an ROS-scavenging agent, 10 mM *N*-acetyl cysteine (NAC) was amended.

### Time-to-Kill Experiment

The MRSA bacteria were cultured to OD_600_ of 1.0 in an MH broth and then diluted to approximately 5 × 10^5^ CFU/ml. The cell cultures were treated with 0.78, 1.56, 3.13, 6.25, and 12.5 μg/ml of the hit (c5) with more potent antibacterial activity and incubated for 10 h at 37°C. At 1 or 2-h time intervals, the aliquots (1 ml) were taken from the samples and then plated onto LB agar plates to enumerate the viable bacteria. The experiments were performed four times.

### Cell Permeabilization Using Polymyxin B (PMB)

*Escherichia coli* and *P. aeruginosa* cells were grown until the logarithmic phase in LB medium and then diluted to 5 × 10^5^ CFU/ml. To increase the membrane permeability, the cells were treated with 0.05, 0.1, or 0.2 μg/ml of PMB. The samples were collected at various time intervals and then plated onto LB agar plates to enumerate the viable bacteria. The experiments were performed four times.

### Measurement of Antibacterial Efficacy

In order to determine the antibacterial efficacy of c5, *Drosophila* systemic infection was performed as previously described (Lau et al., 2003; Kim et al., 2008). *Drosophila melanogaster* strain Oregon R, was grown and maintained at 25°C using the corn meal-dextrose medium [0.93% agar, 6.24% dry yeast, 4.08% corn meal, 8.62% dextrose, 0.1% methyl paraben, and 0.45% (v/v) propionic acid]. For systemic infection, 4- to 5-day-old adult female flies were infected by pricking the dorsal thorax with a 0.4 mm needle (Ernest F. Fullam, Inc.). The needle was dipped into PBS-diluted bacterial suspension containing PA14 (10^7^ CFU/ml) and/or SA3 (10^8^ CFU/ml) grown to the OD_600_ of 3.0 (Lee et al., 2018). Infected flies were transferred to a new medium overlaid with 80 μl of c5 compound (1 mg/ml). Survival rates of the infected flies were monitored for up to 72 h post-infection. Flies that died within 12 h were excluded in mortality determination. Mortality assay was repeated at least four times.

### Spontaneous Mutagenesis and Whole-Genome Sequencing (WGS)

*Staphylococcus aureus* SA3 was grown at 37°C using LB broth. Overnight-grown cultures were used as inoculum (1.6 × 10^7^ CFU/ml) into fresh LB broth and grown at 37°C in a shaking incubator to OD_600_ of 1.0. The cell culture was plated onto LB agar plates with 40 μg/ml of c5 and incubated at 37°C for approximately 36 h. The well-growing colonies were isolated and analyzed by WGS.

### Respiration Activity Assay Using 2,3,5-Thriphenyl Tetrazolium (TTC)

The bacterial cells were grown to the logarithmic growth phase (OD_600_ of 1.0) at 37°C. Three microliters of cell cultures were spotted onto an LB agar plate containing either TTC (0.015%) or TTC (0.015%) and glucose (0.2%).

### Measurement of ROS Generation

Reactive oxygen species generation was measured using hydroxyphenyl fluorescein (HPF), which is known to react with hydroxyl radicals and peroxynitrite (Setsukinai et al., 2003). The bacteria were cultured to OD_600_ of 0.5 in M9 minimal medium and incubated with HPF (5 μM) for 30 min. The cells were then washed and resuspended in 1 ml of M9 minimal medium containing 0.75 and 1.5 μg/ml of c5 or b4. The samples were incubated for 3 h at 37°C and the fluorescence was measured at a 1-h time interval at 490-nm excitation and 525-nm emission. ROS measurement was repeated three times.

### Statistics

Statistical analysis was performed using the GraphPad Prism version 6.0 (GraphPad Software, La Jolla, CA, United States). Data for each analysis represents a set of five repetitions. Statistical significance between the groups is indicated, based on a *p*-value of less than 0.01 (^∗^*p* < 0.01; ^∗∗^*p* < 0.001) by using the Kaplan-Meier log-rank test and the Student’s *t*-test. Error bars represent the standard deviations.

## Results

### YM155 and Its Congeners Display Antibacterial Activity

**FIGURE 1:**
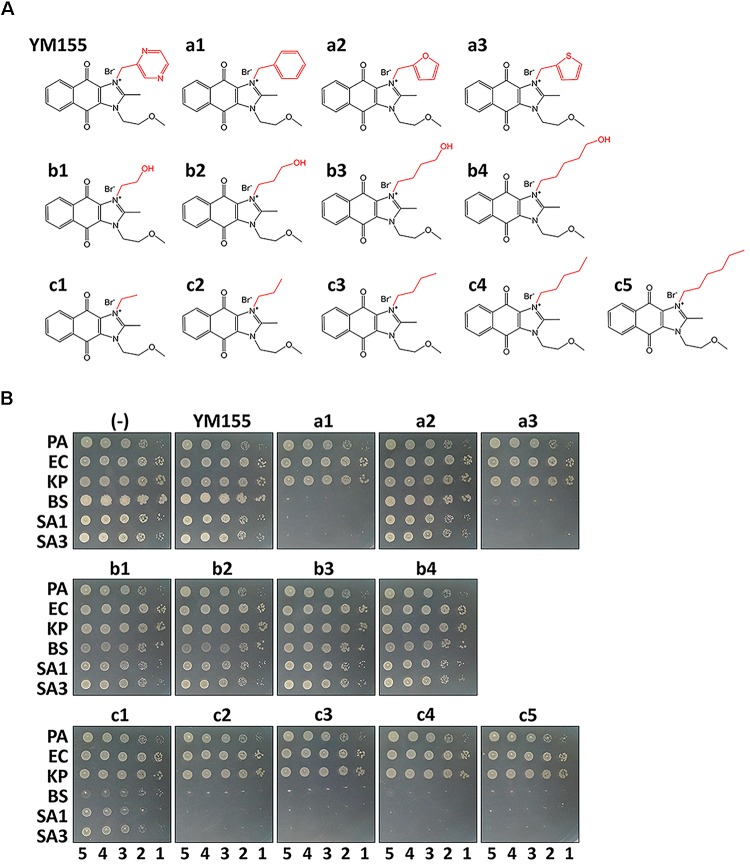
Antibacterial activity *in vitro*. **(A)** Structure of YM155 and its analogs: Group a (a1∼a3), b (b1∼b4) and c (c1∼5) are analogs at N3 position of YM155. Group a has a ring structure, group b has a terminally hydroxylated alkyl chain, and group c has an alkyl chain. **(B)** Susceptibility of various bacterial strains to YM155 and its analogs: The Gram-negative (*Pseudomonas aeruginosa*, PA; *Escherichia coli*, EC; and *Klebsiella* *pneumoniae*, KP) and Gram-positive (*Bacillus subtilis*, BS; *Staphylococcus aureus* MSSA, SA1; and *S. aureus* MRSA, SA3) cells were grown to the logarithmic growth phase. Ten-fold serial dilutions of the cell cultures were spotted onto an LB agar plate (–), and LB agar plate containing YM155 (7.5 μg/ml), or its analogs (7.5 μg/ml). The numbers indicate the log(CFU) of the applied bacterial spots.

During our endeavors to find potential antibacterial against the major nosocomial bacterial pathogens, we identified the weak antibacterial activity of YM155 against a methicillin-resistant *S. aureus* strain, SA3 (Table 1). We designed its analogs at the N3 position, and a series of compounds depicted in Figure 1A were tested for antibacterial activity in comparison with YM155. Group a (a1∼a3) has an aromatic or heteroaromatic ring structure and group b (b1∼b4) with a terminal hydroxyl residue, includes b1 and b2, both of which have a similar structure of half of the pyrazine ring of YM155. Group c (c1∼c5) was similar to group b, but without the hydroxyl residue. We excluded group a compounds due to the lack of aqueous solubility. Among the group b compounds, b1 showed the highest antibacterial activity, whereas b3 displayed weaker activity than YM155 did (Figure 1B and Table 1). It is of marked interest that group c compounds exhibited the strongest antibacterial activity, depending on the length of the chains. The time-dependent bactericidal activity of c5 was also determined (Supplementary Figure S1). These results suggest that the substitution at the N3 position is critical to modulate the activity of the YM155 scaffold in regards to the antibacterial activity against Gram-positive bacteria including MRSA.

**Table 1:** MICs of YM155 and its analogs against MRSA, SA3^a^.

Compound	MIC (μg/ml)	MIC (μM)	MW
YM155	50	112.79	443.29
a1	3.13	7.09	441.32
a2	100	231.87	431.28
a3	6.25	13.97	447.35
b1	12.5	31.63	395.25
b2	25	61.09	409.27
b3	50	118.12	423.3
b4	25	57.17	437.33
c1	25	65.91	379.3
c2	12.5	31.78	393.3
c3	12.5	30.69	407.3
c4	6.25	14.84	421.3
c5	3.13	7.19	435.3


*a: the MIC values of these compounds against *Escherichia coli* (MG1655) and *Pseudomonas aeruginosa* (PA14) have been determined as >500 μg/ml.*

### Bioactivity of c5, a Representative YM155 Congener, Is Selective

**FIGURE 2:**
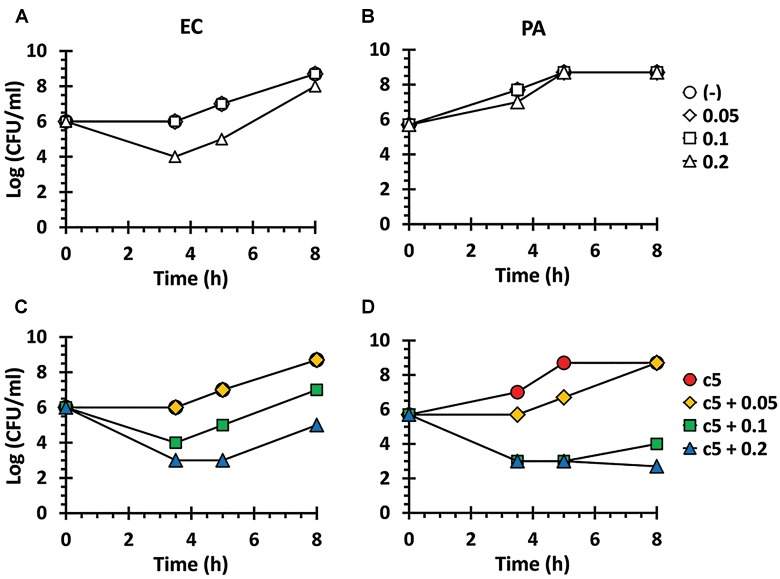
Antibacterial activity in the presence of polymyxin B (PMB). Antibacterial activity against *E. coli* (EC) **(A,C)** and *P. aeruginosa* (PA) **(B,D)** in the presence of PMB. The EC and PA culture suspensions (5 × 10^5^ CFU/ml) were incubated in LB broth with nothing (open circle), with c5 (12.5 μg/ml) only (filled circle), with PMB (0.05, 0.1, or 0.2 μg/ml) only **(A,B)**, or with both c5 (12.5 μg/ml) and PMB (0.05, 0.1, or 0.2 μg/ml) **(C,D)**. The viable cells were counted by plating onto LB agar plates at the designated time points.

As shown in Figure 1B, these compounds were not sufficiently antibacterial against Gram-negative bacteria, suggesting that the outer membrane might prevent these compounds from entering the bacterial cells as observed with other antibacterials such as erythromycin and fusidic acid (Saha et al., 2008; Delcour, 2009). Thus, we have examined whether the permeabilizing agent, polymyxin B (PMB), at a sub-inhibitory concentration, could affect the growth of the Gram-negative bacteria in the presence of c5 (Figure 1A), as the representative compound with the lowest MIC and good aqueous solubility (Table 1). As shown in Figure 2, PMB did not affect the growth of *E. coli* (MG1655) and *P. aeruginosa* (PA14) below 0.1 and 0.2 μg/ml, respectively. At the sub-lethal concentrations of PMB, c5 (12.5 μg/ml) was able to kill both Gram-negative species in a dose-dependent manner of PMB, whereas their growth was not affected at all in the absence of PMB (Figure 2C,D). Interestingly, we have observed the similar but slightly different killing patterns using the PMB nonapeptide (PMBN), a non-toxic derivative of PMB (Supplementary Figure S2), suggesting the presumable synergy between PMB and c5 in ROS generation in Gram-negative bacteria (see below). These results suggest that the selective antibacterial activity of c5, and presumably the aforementioned congeneric compounds, against the Gram-positive bacteria, is most likely associated with the permeability to the target bacterial cells.

### c5 Rescues *Drosophila* Specifically From Infection Caused by MRSA

**FIGURE 3:**
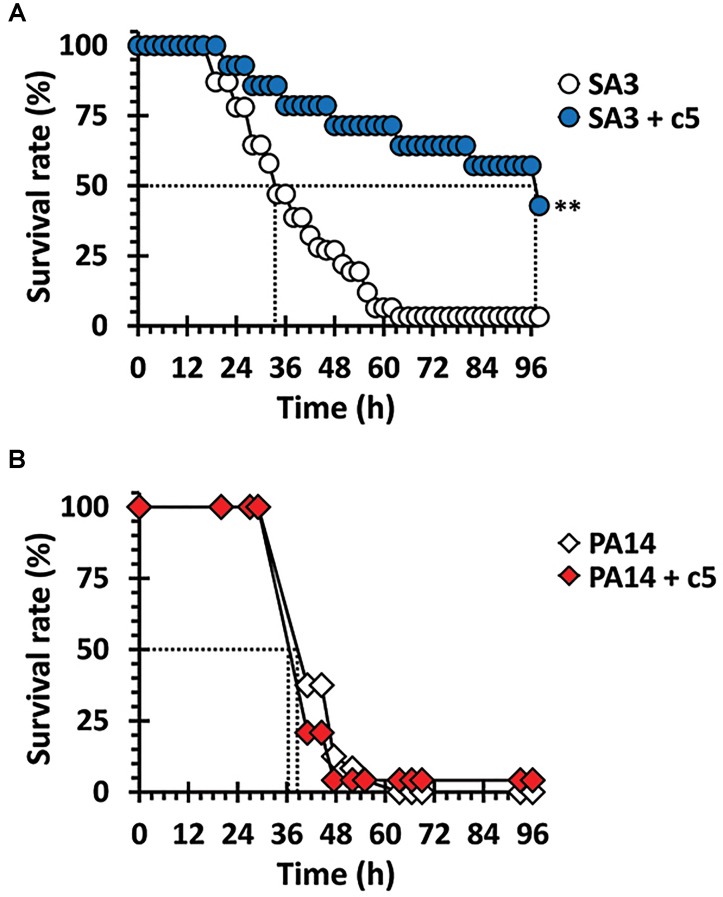
Antibacterial efficacy *in vivo*. Mortality of infected flies fed with c5 was measured. Infected flies with SA3 **(A)** and PA14 **(B)** were transferred to a new medium overlaid with 1 mg/ml of c5 (filled) or without treatment (open). The dotted lines represent the time required to reach 50% mortality. The statistical significance based on a log-rank test is indicated as follows: ^∗∗^, *p* < 0.001.

The antibacterial efficacy of c5 was evaluated in the *Drosophila* systemic infection model. The MRSA, SA3 with multiple antibiotic resistance was more virulent than the methicillin-sensitive *S. aureus* (MSSA), SA1 in the fly systemic infection model (Supplementary Figure S3). Based on the antibacterial activity of c5 toward SA3, flies were pricked with SA3 or *P. aeruginosa* PA14 as described in Materials and Methods. Figure 3 shows that a significant protection from mortality, caused by SA3, was observed by adding the c5 compound to the fly media (up to 1 mg/ml), whereas c5 did not reduce the mortality caused by PA14. This is also important with regards to the toxicity of this compound, in that c5 is not apparently toxic to the flies. This needs to be further verified using mammalian acute and chronic toxicity models. Altogether, we suggest that the potent *in vitro* activity of c5 translates *in vivo* into a selective antibacterial activity that rescues flies from the systemic infection caused by MRSA, but not by *P. aeruginosa*.

### The Mutants in Quinone Biosynthesis Are Resistant to c5

**FIGURE 4:**
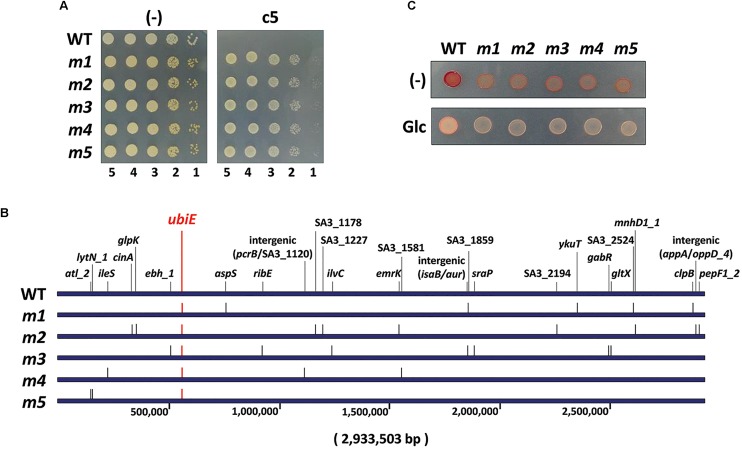
Antibacterial resistance. **(A)** Resistance of spontaneous mutants (*m1*∼*m5*) to c5. The wild type (WT) SA3 and its c5-resistant mutant (*m1*∼*m5*) cells were grown to the logarithmic growth phase, and then ten-fold serial dilutions from the cells were spotted onto an LB agar plate (–) and LB agar plate containing 5.0 μg/ml of c5. **(B)** Schematic representation of the mutation genes on the genome coordinates of the c5-resistant mutants (*m1*∼*m5*) in comparison with the 2,933,503-bp SA3 genome. The number indicates the relative nucleotide position of each genome. **(C)** Respiration activity of the c5-resistant mutants (*m1*∼*m5*) using 2,3,5-thriphenyl tetrazolium (TTC). The logarithmic growth phase cells of the wild type (WT) and c5-resistant mutant (*m1*∼*m5*) cells were spotted onto an LB agar plate containing 0.015% of TTC (–) and an LB agar plate containing 0.015% of TTC and 0.2% of glucose (Glc).

In order to assess the mode of action of the antibacterial activity of c5, we have isolated 11 spontaneous mutants (*m1*∼*m11*) whose growth was not inhibited by c5 (Figure 4A). The frequency of the spontaneous mutation was about 10^9^, within the normal range of spontaneous mutations due to base transition. Whole genome sequencing of the randomly selected five c5-resistant mutants (*m1*∼*m5*) revealed multiple mutations in each mutant (6 for *m1*, 10 for *m2*, 8 for *m3*, 4 for *m4*, and 3 for *m5*) (Figure 4B). It is remarkable that they had nonsense mutations in common for a gene, *ubiE*: TTA to TAA at the 54th leucine codon for *m1*∼*m4* and AAA to TAA at the 52nd lysine codon for *m5*. This gene encodes a methyltransferase crucial for biosynthesis of ubiquinone and menaquinone (MQ), which are the widespread respiratory quinones in bacterial species. This result suggests that a certain change caused by the *ubiE* mutations in the respiratory chain might be able to render MRSA resistant to c5. Because nonsense mutations normally result in a loss-of-function, it is highly likely that UbiE is not the direct target for c5 and that the change in respiratory chain(s) due to the UbiE loss may affect the electron flow during the respiration, resulting in the reduction of the c5 bioactivity. This result suggests that the *ubiE* mutations of the aforementioned mutants might most likely be responsible for the c5-resistance phenotype. Alternatively, it is also probable that a change, due to the mutations in quinone biosynthesis, may enable the cells to bypass the c5-inhibited pathway for growth, which needs to be further elucidated.

### Antibacterial Activity of c5 Involves Reactive Oxygen Species (ROS)

**FIGURE 5:**
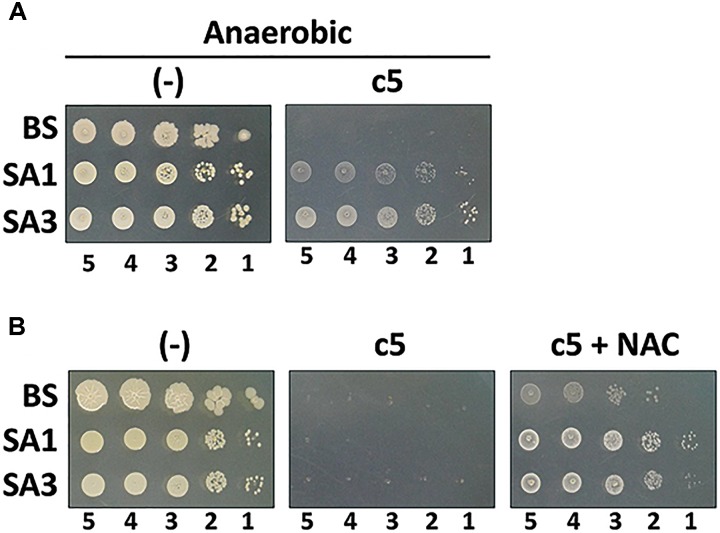
Antibacterial activity under ROS-compromising conditions. **(A)** Antibacterial activity of c5 under anaerobic condition. The Gram-positive bacterial cells (*B. subtilis*, BS; *S. aureus* MSSA, SA1; *S. aureus* MRSA, SA3) were grown to the logarithmic growth phase. Ten-fold serial dilutions from the cell cultures were spotted onto an LB agar plate (–) and an LB agar plate containing 5.0 μg/ml of c5. **(B)** Antibacterial activity of c5 in the presence of *N*-acetylcysteine (NAC) under aerobic condition. The bacterial cells in A were grown to the logarithmic growth phase. Ten-fold serial dilutions from the cell cultures were spotted onto an LB agar plate (–) and LB agar plate containing 5.0 μg/ml of c5 with (c5+NAC) or without (c5) 10 mM NAC. The numbers indicate the log(CFU) of the applied bacterial spots.

**FIGURE 6:**
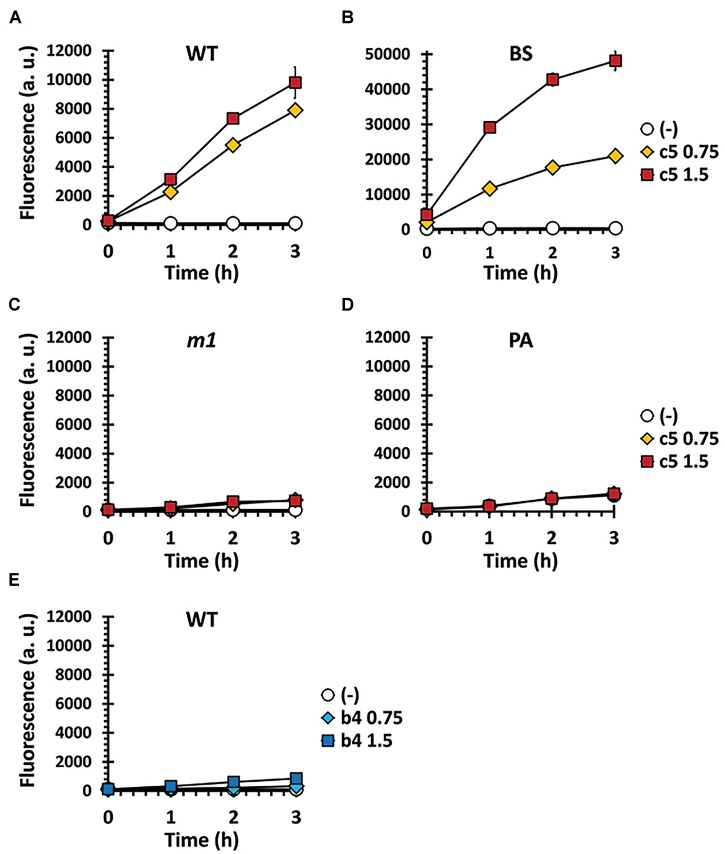
ROS formation by c5 under aerobic condition. ROS generation was measured in SA3 (WT) **(A,E)** and its mutant (*m1*) **(C)** as well as in *B. subtilis* (BS) **(B)** and *P. aeruginosa* (PA) **(D)**, which had been grown to the logarithmic growth phase in M9 minimal medium containing 0.75 (diamond) or 1.5 μg/ml (square) of either c5 **(A–D)** or b4 **(E)**. No-compound controls (empty circle) were included to investigate the endogenous ROS generation. The data of three independent experiments were pooled and means are shown. Error bars represent standard deviations of the means.

c5, as well as the other compounds in this study, contain the naphthoquinone scaffold of vitamin K-family compounds such as MQ (vitamin K_2_) and menadione (MD, vitamin K_3_). MQ is the major or sole isoprenoid quinone in most Gram-positive and anaerobic Gram-negative bacteria, which transfer electrons in the respiratory chain (Collins and Jones, 1981), while MD is a synthetic chemical well-known for its reactive oxygen species (ROS)-generating activity (Criddle et al., 2006). More importantly, the *ubiE* mutations that may affect the electron flow in the respiratory chain, are involved in c5-resistance (Figure 4). We hypothesized that ROS generation during aerobic respiration might be facilitated and/or augmented by c5. In order to verify this, we first assessed the antibacterial activity under anaerobic conditions (Figure 5A). The antibacterial activity against *S. aureus* strains was compromised under anaerobic conditions. To further verify the involvement of ROS generation in the c5 bioactivity, an antioxidant compound, *N*-acetylcysteine (NAC) was exploited. As shown in Figure 5B, the antibacterial activity of c5 was also impaired in the presence of NAC under aerobic conditions. We next investigated whether ROS generation was indeed observed by c5 treatment in SA3, using the hydroxyl radical sensor, hydroxyphenyl fluorescein (HPF). As shown in Figure 6, ROS generation was clearly observed in the Gram-positive bacteria (SA3 and BS). It is noteworthy that the c5-mediated ROS generation was impaired in a c5-resistant mutant as well as in *P. aeruginosa*. Unlike c5, b4, as a representative with a similar-length side chain but without antibacterial activity (Figure 1), was clearly unable to generate ROS at similar concentrations (Figure 6E). These results suggest that the antibacterial activity of c5 is most likely associated with ROS generation that would be enhanced by this compound in MRSA.

## Discussion

Methicillin-resistant *Staphylococcus aureus* infections continue to pose a new threat and is a significant public health challenge, due to over 50% failure rates in the conventional antibiotic treatment of infected patients (Soriano et al., 2008; Gould et al., 2012). Despite the urgent need for new effective antibacterials to treat MRSA infections, the traditional approach in drug development is consuming tremendous amounts of time and resources, as bacterial resistance continues to rapidly develop. This has led researchers to explore alternative approaches such as drug repurposing, which utilizes approved drugs used primarily for other purposes to treat bacterial infections. Auranofin is such an example, as it was initially approved as an anti-rheumatic agent. It is known that auranofin displays antiviral, antiprotozoal, and anticancer activities (Chirullo et al., 2013; Chen et al., 2014). It also showed potent antibacterial activity against replicating and non-replicating *Mycobacterium tuberculosis* as well as other Gram-positive bacteria including MRSA (Cassetta et al., 2014; Harbut et al., 2015).

As an independent approach, we started with a different compound, YM155, which is an anticancer drug candidate. YM155 is currently under clinical trials for combination therapy with approved anticancer drugs such as carboplatin and paclitaxel (Kelly et al., 2013). The fused imidazolium compound possesses the naphthoquinone scaffold that might act as the pharmacophore which presumably provokes ROS formation. These result in mitochondrial dysfunction and subsequent apoptosis, the major contributor of bioactivity in this compound (Ho et al., 2015). This YM155 property has led us to the idea that YM155 may exhibit bactericidal activity upon entry into the bacterial cells. In the present study, we observed weak or cryptic antibacterial activity of YM155 against Gram-positive bacteria including MRSA. To witness this observation, we sought to enhance its antibacterial activity and solubility through chemical modification at the N3 position of the scaffold. This position is relatively easy to introduce several modifications at a reasonable efficiency, in a late stage of chemical synthesis. More importantly for this study, pyrazine moiety that is not easy to deal with in chemical synthesis, was required for anticancer activity, but not for antibacterial activity. Among the chemically modified congeners, those with alkyl chains displayed better activity and solubility, and the c5 compound with a hexyl chain was selected for further analyses as the antibacterial hit compound for anti-MRSA candidates. Based on these considerations, our approach clearly differs from drug repurposing studies, since included chemical modification(s) to change (i.e., redirect) the target indications. This new approach in drug discovery would be “drug redirecting.” The redirected drugs literally belong to the incrementally modified drugs (IMDs).

Another key implication of this study was that our drug redirecting strategy was validated by using the naphthoquinone scaffold as the pharmacophore, fused with the imidazolium ring, as the moiety for chemical modification affordable to modulate its target-specific permeability. Currently, we have been more comprehensively investigating whether the N3 position of this scaffold is necessary and sufficient to affect the target-specificity. This investigation is necessary in order to identify the molecular and submolecular determinants of the target cells, primarily by massively modulating the permeability. This knowledge will be exploited for application of the naphthoquinone imidazolium scaffold and its ROS-generating activity, to inhibit various cell types associated with human diseases including mammalians, protozoa, fungi, and Gram-negative bacteria. With the recent discovery of the key traits required for the permeability to Gram-negative bacteria (Richter et al., 2017), more comprehensive structure-activity-relationship (SAR) studies on this scaffold will further strengthen the utility of the drug platform, for more suitable exploitation by rewiring or broadening the target-specificity. In a broader context, however, we believe we are now in the beginning stage of reincarnating the approved drugs or known compounds based on chemical modifications beyond just repurposing them as they are. This drug redirecting can be done primarily by structural modifications to revise their target cell types. In this case, their pharmacophores essentially need to work irrespective of cell types, once they enter the subcellular localizations such as the cytosol and cytoplasmic membrane. While we have demonstrated that the fused naphthoquinone imidazolium bromide works well for drug redirecting strategies in antibacterials, further research is required to reveal additional pharmacophores appropriate for drug redirecting strategies and to improve the utility of this new approach. All these endeavors will be valuable in the era of target-specific and/or side effect-free therapy, as well as in new antibacterial discovery, since we need to target the specific sets of harmfully outgrowing microbes among the complex microbial communities in our bodies.

## Author Contributions

Y-HC and I-YC conceived and designed the study. H-JJ, I-YC, CL, and SC designed and performed the experiments, and collected and analyzed the experimental data. CL, SC, and S-HK synthesized and identified the chemical compounds. B-oK and EK provided reagents. H-JJ, I-YC, S-HK, and Y-HC wrote the manuscript. All authors reviewed the manuscript.

## Conflict of Interest Statement

The authors declare that the research was conducted in the absence of any commercial or financial relationships that could be construed as a potential conflict of interest.
