# Supplementary material for: Redirecting an Anticancer to an Antibacterial Hit Against Methicillin-Resistant Staphylococcus aureus
Source: Front Microbiol. 2019 Feb 25;10:350. doi: 10.3389/fmicb.2019.00350 (PMC6398426; doi:10.3389/fmicb.2019.00350)
Supplement: Supplementary file 1 [file Data_Sheet_1.pdf]

SUPPORTING INFORMATION

Redirecting an anticancer to an antibacterial hit against methicillin-resistant *Staphylococcus aureus*

Hye-Jeong Jang,† In-Young Chung,† Changjin Lim, Sungkyun Chung, Bi-o Kim, Eun Sook Kim, Seok-Ho Kim,* and You-Hee Cho*

Department of Pharmacy, College of Pharmacy and Institute of Pharmaceutical Sciences, CHA University, Gyeonggi-do 13488, Korea

*Corresponding authors.
Phone: 82-31-881-7169 (S.-H.K.) and 82-31-881-7165 (Y.-H.C)
Electronic mail: ksh3410@cha.ac.kr (S.-H.K.) and youhee@cha.ac.kr (Y.-H.C)

†H.-J. Jang and I.-Y. Chung contributed equally to this work.

Running Title: Drug redirecting from anticancer to antibacterial

Keywords: MRSA, Gram-positive, antibacterials, drug repurposing, drug redirecting

TABLE S1. Bacterial strains in this study

Strain	***Relevant characteristics or purpose***[a]	***Reference or source***
SA1	*Staphylococcus aureus* Newman	(Baba et al., 2008)
SA3	*S. aureus* MRSA laboratory strain; Mc^R	This study
m1	Spontaneous c5-resistant mutant of SA3; Mc^R	This study
m2	Spontaneous c5-resistant mutant of SA3; Mc^R	This study
m3	Spontaneous c5-resistant mutant of SA3; Mc^R	This study
m4	Spontaneous c5-resistant mutant of SA3; Mc^R	This study
m5	Spontaneous c5-resistant mutant of SA3; Mc^R	This study
PA14	*Pseudomonas aeruginosa* laboratory strain; Mc^R, Rif^R	(Rahme et al., 1995)
pqsA	PA14 with in-frame deletion of *pqsA*; Mc^R, Rif^R	(Kim et al., 2010)
EC	*Escherichia coli* EDL933; Mc^R	(Strockbine et al., 1986)
KP	*Klebsiella pneumoniae* KP1; Mc^R	This study
BS	*Bacillus subtilis* PS832	(Tovar-Rojo et al., 1991)

a : Mc^R, methicillin-resistant, Rif^R, rifampicin-resistant

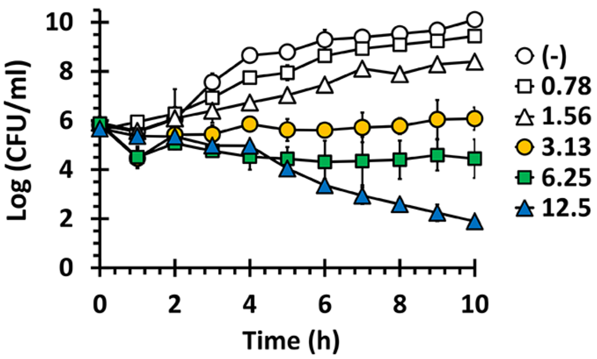

FIGURE S1. Time-to-kill assay. Antibacterial activity of c5 against the MRSA, SA3. Time-to-kill assays were performed with 5 × 10^5 CFU/ml of SA3. The cell suspensions were treated with various concentrations of c5 (0, 0.78, 1.56, 3.13, 6.25, and 12.5 µg/ml). The experiment was conducted for 10 h at 37°C. At the designated time points, the samples were collected and then the survivor cells were enumerated by viable cell counts.

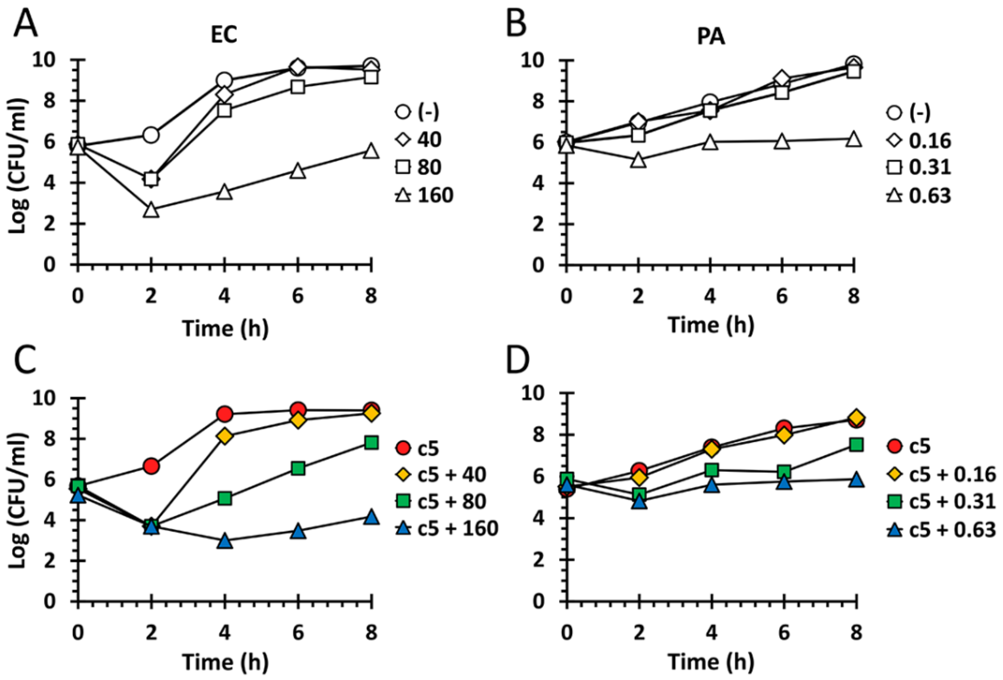

FIGURE S2. Antibacterial activity in the presence of polymyxin B nonapeptide (PMBN).

Antibacterial activity against *E. coli* (EC) (A and C) and *P. aeruginosa* (PA) (B and D) in the presence of PMBN. The EC and PA culture suspensions (5 × 10^5 CFU/ml) were incubated in LB broth with nothing (open circle), with c5 (12.5 μg/ml) only (filled circle), with PMBN only (A and B), or with both c5 (12.5 μg/ml) and PMBN (C and D). The numbers are PMBN concentrations: 40, 80, and 160 μg/ml for EC (A and C); 0.16, 0.31, and 0.63 μg/ml for PA (B and D). The viable cells were counted by plating onto LB agar plates at the designated time points.

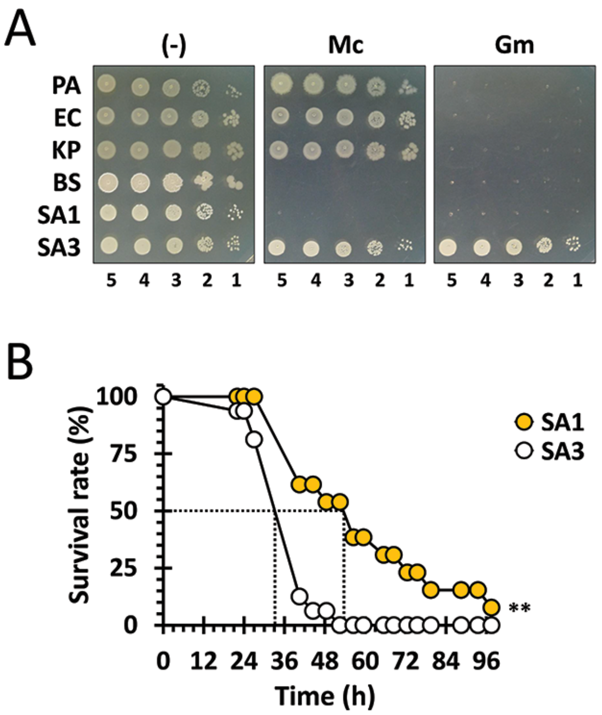

FIGURE S3. Antibiotic resistance and *Drosophila* virulence of the bacterial strains. (A) Susceptibility of various bacterial strains to antibiotics. The representative Gram-negative (*P. aeruginosa*, PA; *E. coli*, EC; and *K. pneumoniae* KP) and Gram-positive (*B. subtilis*, BS; *S. aureus* SA1 (MSSA) and SA3 (MRSA)) cells were grown to logarithmic growth phase. Ten-fold serial diluted cells were spotted onto an LB agar plate, and LB agar plate containing methicillin (50 μg/ml) (Mc), or gentamicin (50 μg/ml) (Gm). The numbers indicate the log(CFU) of the applied bacterial spots. (B) Mortality of SA1- or SA3-infected flies. Groups of 40 flies were infected with either SA1 or SA3 as described in Methods. Mortality curves were determined, based on the survivals in infected flies. The dotted lines represent the time required to reach 50% mortality. The statistical significance based on a log-rank test is indicated as follows: **, $p < 0.001$.

REFERENCE

Baba, T., Bae, T., Schneewind, O., Takeuchi, F., and Hiramatsu, K. (2008). Genome sequence of *Staphylococcus aureus* strain Newman and comparative analysis of *staphylococcal* genomes: polymorphism and evolution of two major pathogenicity islands. *J Bacteriol* 190, 300-310. doi: 10.1128/JB.01000-07

Kim, K., Kim, Y. U., Koh, B. H., Hwang, S. S., Kim, S. H., Lepine, F., et al. (2010). HHQ and PQS, two *Pseudomonas aeruginosa* quorum-sensing molecules, down-regulate the innate immune responses through the nuclear factor-kappaB pathway. *Immunology* 129, 578-588. doi: 10.1111/j.1365-2567.2009.03160.x

Rahme, L. G., Stevens, E. J., Wolfort, S. F., Shao, J., Tompkins, R. G., and Ausubel, F. M. (1995). Common virulence factors for bacterial pathogenicity in plants and animals. *Science* 268, 1899-1902. doi: http://www.ncbi.nlm.nih.gov/pubmed/7604262

Strockbine, N. A., Marques, L. R., Newland, J. W., Smith, H. W., Holmes, R. K., and O'Brien, A. D. (1986). Two toxin-converting phages from *Escherichia coli* O157:H7 strain 933 encode antigenically distinct toxins with similar biologic activities. *Infect Immun* 53, 135-140. doi: http://www.ncbi.nlm.nih.gov/pubmed/3522426

Tovar-Rojo, F., and Setlow, P. (1991). Effects of mutant small, acid-soluble spore proteins from *Bacillus subtilis* on DNA in vivo and in vitro. *J Bacteriol* 173, 4827-4835. doi: http://www.ncbi.nlm.nih.gov/pubmed/3522426
